# Annual Meeting of the American Gynæcological Society

**Published:** 1878-11

**Authors:** 


### Annual Meeting of the American Gynaecological Society.

The third annual meeting of the American Gynæcological Society was held at the hall of the College of Physicians, Philadelphia, September 25th, 26th, and 27th, the sessions lasting each day from ten to one and from three to five o'clock. The following members were present: Fordyce Barker, T. A. Emmett, T. G. Thomas, E. Noeggerath, P. F Mundé, of New York; J. Byrne, H. J. Garrigues, of Brooklyn; J. D. Trask, of Astoria, L. I.; D. H. Storer, G. H. Lyman, A. D. Sinclair, J. R. Chadwick, W. L. Richardson, of Boston; Gilman Kimball, of Lowell; W. Goodell, R. A. F. Penrose, A. H. Smith, T. M. Drysdale, E. Wilson, of Philadelphia; S. C. Busey, J. T. Johnson, of Washington; H. P. C. Wilson, of Baltimore; J. P. White, of Buffalo; W. H. Byford, of Chicago; J. C. Reeve, of Dayton, Ohio; H. F. Campbell, of Augusta, Ga.; and G. J. Engelmann, of St. Louis.

A number of medical gentlemen present were made guests of the society, and Dr. Barker, the ex-president, and Dr. J. L. Atlee, of Lancaster, Pa., an honorary member, were requested to take seats upon the platform.

WEDNESDAY, morning session The senior vice-president, Dr. William Goodell, called to society to order, and introduced Dr. A. H. Smith, who gave the address of welcome in behalf of the Philadelphia members.

Dr. J. C. Reeve read an account of a case of rupture of the perinæum without any implication of the vulva. The patient was a multipara, confined under the care of a midwife. Both anal sphincters were ruptured, and the child was delivered through the anus. In the discussion which followed, Dr. Campbell dwelt on the importance of an immediate operation in cases of rupture, since the lochia and other discharges do not seem in any way to interfere with the healing; while it often happens that operations performed later, the edges of the laceration being refreshed, fail. Dr. White thought that greater stress should be placed on the necessity of preventing these lacerations. He strongly recommended the making of lateral incisions. In this way the perinæum may be preserved intact, and such incisions very rapidly heal, the edges being brought together by the natural contraction of the parts.

A paper by Dr. Marion Sims on the Surgical Treatment of Dysmenorrhœa was then read by the secretary. The object of the paper was to establish the author's claim to having first suggested the antero-posterior incisions of the cervix in cases of stenosis, and the great value which he attached to this operation.

Dr. Barker thought it at least remarkable that one gynæcologist should have performed this operation a thousand times, while others, having equal opportunities for operating, should so rarely see cases which seemed to call for such treatment. He was convinced that the operation was often performed unnecessarily and injudiciously. He was cognizant of sixteen deaths which had followed this method of treatment, even where the operation had been performed by skillful surgeons. There were many cases of stenosis where some treatment seemed necessary to relieve either a dysmenorrhœa or sterility. He question, however, whether successful results were so often obtained as were reported. He had himself seen over a hundred cases in which an incision had been previously made by operations, but no relief had followed.

Dr. E. Wilson was opposed to this method of treatment, and believed that better results were obtained from a dilatation of the cervix.

Dr. Noeggerath said he could never forget Dr. Peaslee's two objections to this operation. In the first place, in cases of anteflexion of the second degree the canal can be straightened only by an incision running so high up as to open the peritoneal cavity. In the second place, by such a procedure, the parenchymatous structure of the uterus is so injured that there is great danger of septicæmia. There may be cases in which a slight incision, not deeper than one fifth of an inch at the inner os and one fourth of an inch at the outer, may be justifiable. He believed, however, that the vast majority of cases reported as cured were in reality only relieved for two or theree months. Before cases are reported as successful they must be under observation a long time.

Dr. H. P. C. Wilson had no doubt that the operation was too frequently performed, but still there were many cases where it was the proper thing to do. He had seen the most serious results follow the introduction of a sponge tent for the purpose of dilatation.

Dr. Lyman believed strongly in dilatation, and was opposed to the treatment recommended by the author of the paper.

Dr. Emmett said that careful observation had taught him that where the flexion was above the vaginal junction a proper examination of the case would discover trouble elsewhere, and that the uterine difficulty was merely the exponent of some abnormal condition lying outside the uterus itself. It was due to an obstruction of the circulation. A rectal examination will detect a thickening of the broad ligament on one or both sides, the result of a previous cellulitis. The fault, therefore, does not lie in the uterus in the beginning, nor will any such operation effect a cure. He believed firmly that mechanical dysmenorrhœa was a myth. In all these cases where such an operation is contemplated, he urged strongly the searching for the results of a previous cellulitis. Were this done the operation would soon come to be a thing of the past.

Dr. J. P. White reported a case of extra-uterine pregnancy in which the fœtal bones had been discharged through the bladder. Drs. Atlee and Storer reported other cases of extra-uterine pregnancy. The general sentiment was in favor of abstaining from an operation as long as possible, and when surgical interference was demanded then to remove the fœtus, leaving the placenta behind to take care of itself.

Afternoon session. Dr. J. T. Johnson reported a very rare case, in which he was called upon to deal with a combined foot and head presentation, and in which there occurred a fracture of the spine in utero.

Dr. Engelman gave the details of a case in which a child was born with a great many of the bones broken, some of them presenting evidences of having been broken but reunited.

Dr. Noeggerath had seen one case in which there had occurred in utero a fracture of a humerus and also of a femur. Before the child was five years of age thirty-two spontaneous fractures of different bones had taken place.

Dr. Campbell had seen two children, in each of them of whom a fractured rib had been discovered soon after birth.

Dr. Emmett read a most carefully prepared table, giving an analysis of one hundred and sixty-one cases in which he had operated for vesico-vaginal fistula, with an account of the character of the preceeding methods of delivery in each case. He stated that he had never seen a case of vesico-vaginal fistula which had been caused by the use of instruments. In the great majority of cases a neglect to empty the bladder is found to be the exciting cause. He would impress on the minds of the members of the society the necessity in all cases of introducing the catheter, no matter what the patient's statement as to the supposed condition of the bladder may be. The fistulæ in most of these cases had been caused by prolonged pressue.

Dr. Smith thought that the accident was not due to the amount or violence of the pressure, but to the duration of it.

Dr. Storer did not believe in the too frequent use of forceps. If the head ceases to recede, or, in other words, it becomes impacted, then of course they must be used.

Dr. Barker observed that an over-distended bladder was *per se* a cause of retarded labor. The accessory muscles were not brought into action, owing to the pain occasioned by the distention. In cases where the parts are subjected to prolonged pressure we incur the danger of peritonitis, cystitis, and other inflammatory diseases. A prolonged first stage of labor, owing to the depression and exhaustion it produces, is a powerful factor in causing a prolonged second stage. In such cases the administration of an opiate to produce sleep, or of quinine in full doses to excite the uterus, was the proper method of treatment.

Dr. Wilson (Baltimore) believed that the use of the forceps should not be considered as an operation, but should be frequently employed with a view to shortening the labor.

Dr. Barker added that the forceps were demanded not only when the head ceases to recede, but when it ceases to advance.

Dr. Atlee thought that forceps should be used for the purposes of relieving pain and cutting the labor short.

In answer to a question of Dr. Smith, Dr. Emmett said that even where the head was pressed firmly against the pelvic brim it could be pushed back often by means of the forceps, and thus the cather could be introduced. If this could not be effected, he believed in tapping the bladder with the aspirator through the abdominal wall, rather than running the risk of trying to extract a head, the bladder being distended.

Dr. Reeve had thus emptied the bladder with the aspirator in one case, with no unfavorable symptom.

Dr. Goodell thought that the tendency of the discussion was to make too light of the operation of forceps. He was of opinion that their proper use required a certain amount, at least, of practice and skill. He considered that many perineal lacerations were due to the improper use of forceps by inexperienced operators. He advocated the removal of the forceps as soon as the head came to the perinæum, unless there were symptoms demanding an immediate delivery.

Thursday, morning session. Dr. Wilson (Baltimore) gave the details of a case in which he had succeeded in stopping a severe post-partum hæmorrhage by scraping the placental surface of the lining membrane of the uterus with the finger-nails, using the hand as a curette, as it were.

Dr. Penrose gave a summary of the methods proposed for treating post-partum hæmorrhage. He closed his paper by strongly urging the use of common vinegar, a means which he had for years employed, and which had never failed at once to arrest the flow of blood.

A general discussion on the proper treatment of post-partum hæmorrhage followed the reading of the two papers.

Dr. White had never used vinegar as thus recommended, but had many times used it as a local styptic in uterine surgery with remarkable success.

Dr. Thomas considered that all cases of post-partum hæmorrhage were due either to uterine inertia, to some influence which prevents a uterus from contracting, although the uterus itself is not at fault, or to some solution of continuity. He believed that nine out of ten cases were due to some mismanagement on the part of the attending physician, who does not properly see to it that a firm uterine contractien takes place after delivery. A small clot, for example, forms, and this gradually increases; the uterine fibres around it become relaxed, and then follows the hæmorrhage. He thought that the third stage should not be considered as the expulsion of the placenta only, but it should be the complete tonic contraction of the uterus, the expulsion of the placenta being merely an epiphenomenon of this stage. The ferric salts were too dangerous ever to be used except as a last resort. What we want is a stimulating application to the interior of the uterus He did not believe that vinegar was a specific, but that alcohol or hot water would do just as well as anything else.

Dr. Atlee strongly favored the immediate introduction of the hand within the uterus.

Dr. Smith dwelt on the necessity of freeing the uterine cavity from clots, and after that he would advise the injection of hot (110° F.) carbolized water.

Dr. Campbell thought it of the utmost importance in all severe cases to raise the foot of the bed, that the brain might be stimulated by the return of blood to the head.

Dr. Engelmann spoke in defense of the use of ferric salts. The danger was that they were not properly used. The uterine cavity should first be cleared completely of clots, and the iron should be applied through a speculum.

Dr. Trask in reply said he did not believe that the iron was of advantage as a styptic, but as an irritant, and as such there were other and safer means at our disposal.

Dr. Chadwick alluded favorably to the subcutaneous injection of ether, which had proved successful in three cases under his care.

Dr. Barker laid especial stress upon the hæmorrhagic diathesis as one of the predisposing causes of post-partum hæmorrhage. It was often found associated with excessive anæmia, and as such required to be treated from a prophylactic stand-point with the appropriate remedies. If the hand is introduced within the uterine cavity, it should be kept there until expelled by uterine contractions. He had, however, in four cases seen a laceration of the os produced by the introduction of the hand within the uterine cavity. This operation was therefore to be avoided if possible.

Dr. Wm. Goodell then delivered the annual address. After paying a graceful tribute to the memory of Drs. Peaslee and Atlee, who had died during the past year, taking a brief review of the work of the last meeting, and making some useful hints as to the future, he passed at once to the consideration of the relation which neurasthenia bears to the diseases of the womb. The address was a firm and scholarly protest against the tendency of meddlesome gynæcology,—the treatment of local uterine effects instead of the great causes which were producing the effects. The essence of many of these diseases lies in the nerves centres, and not in the sexual organs. He warmly advocated the methods of treatment described and followed with such happy results by Dr. Mitchell.

Afternoon session. Dr. Byford read a paper on Dermoid Tumors of the Ovaries. Some later theories as regards their development were subsequently explained by Dr. Noeggerath.

The next paper was by Dr. Richardson, who spoke on the Treatment of the Acute Parenchymatous Nephritis of Pregnancy, with a special reference to the question of the proper time for the induction of premature labor. He believed that while the quality of the urine gives the signal-note of danger, the quantity of urine daily secreted by the patient should be our guide as to the extent of danger. Many cases of nephritis terminate in eclampsia, while others, oftentimes apparently more severe as judged by the symptoms, do not. The key to the problem he thought was to be found in the fact that in the former class of cases the daily quantity of urine grows less and less, while in the latter, although it becomes diminished, it does not at all reach the small amount obtained in the first class of cases. Wherever, then, we have to deal with the acute nephritis of pregnancy, the writer advocated the keeping of a daily record of the quantity of the urine secreted. If this falls below a certain point, despite all efforts to prevent it, then labor should be induced; otherwise it should not, no matter how grave the accompanying symptoms may be.

Drs. Thomas, Barker, and Lyman agreed with the writer, and thought the suggestion an eminently practical one for future investigations.

Friday. The morning session began at nine o'clock with closed doors. The following elections took place:—

President, T. G. Thomas, of New York; vice-presidents, D. H. Storer, of Boston, H. P. C. Wilson, of Baltimore; council, T. A. Emmett, of New York, A. H. Smith, of Philadelphia, John Byrne, of Brooklyn. G. J. Engelmann, of St. Louis; secretary, J. R. Chadwick, of Boston; treasurer, P. F. Mundé, of New York: honorary Fellows, J. S. Billings, U. S. A., Washington, D. C., J. Matthews Duncan, London; active Fellow, Nathan Bozeman, of New York.

It was voted that the next meeting be at Baltimore, the third Wednesday of September, 1878.

Dr. S. C. Busey read an account of a case of Alternating Anterior and Posterior Version of the Uterus.

Dr. Garrigues contributed a valuable historical account of the operation of gastro-elytrotomy, with a critical examination of the merits of the operation.

In the discussion which ensued Dr. Thomas stated that thus far no hæmorrhage had followed the operation. Should it do so, it could easily be controlled by a carbolized tampon in the vagina, and a second in the iliac fossa, the latter being held in position by a firm bandage of sticking-plaster. During the last two hundred and fifty years New York had seen only one successful case of Cæsarean section, while in the last eight years there had been five successful cases of gastro-elytrotomy. There was no danger of creating a urinary fistula; the worst that could happen was a rent in the bladder, and such rents always readily healed.

Dr. Byford favored the operation as being much safer than gastro-hysterotomy.

Dr. Bozeman considered the operation a very dangerous one, owing to the fact that the ureter must necessarily be injured, and a ureto-vaginal fistula was a very serious affair, there being only one case on record of such a fistula being healed.

Both Dr. Thomas and Dr. Garrigues replied that the danger of injury to the ureter was an imaginary one, the latter having demonstrated on the cadaver that the incision and tear are made below and parallel to the normal position of the ureter.

Dr. Smith read an elaborate paper in which he entirely opposed the use of the forceps as a lever, and endeavored to show, by a mathematical demonstration, that the pendulum-like use of the forceps was unsafe, and contrary to the true principles of mechanics.

Afternoon session. Dr. Barker believed that in many cases, while both blades of the forceps should advance at the same time, they should move at different rates. In this way the amount of irritation, friction, and compression is relieved, for you thus allow intervals of rest, now on one side and now on the other of the vaginal track. In no case should one end of the head be thrown back while the other is brought forward, for in that way injury would be done.

Dr. White favored the pendulum use of the forceps under certain restrictions.

Dr. Storer and Dr. Penrose also took exception to the views of the reader.

Dr. Thomas believed that the forceps should be used as tractors when they could, but as levers when they must. If the delivery is to be made after a long labor, when there is œdema from pressure on the venous system, then the lever action must be used, for only in this way can the œdema be gradually overcome. It was the same principle as we should apply in drawing a ring from a finger which was in a normal condition, and again when the finger was swollen. He believed that in all cases in which the pendulum movement was used, it must be as an adjunct only of a simultaneous traction.

Dr. Goodell thought that in gross the ideas of Dr. Smith were correct, but in detail they were wrong. The error was in not remembering that the head was compressible. The leverage must be made always slowly, carefully, and within a limited space.

Dr. Campbell read a paper on Rectal Alimentation in the Nausea and Inanition of Pregnancy.

Dr. Goodell then delivered the farwell address, and after votes of thanks to the secretary and retiring president the society adjourned, to meet in Baltimore.—*Boston Medical Journal.*

